# Electrocardiographic and Echocardiographic predictors of paroxysmal atrial fibrillation detected after ischemic stroke

**DOI:** 10.1186/s12872-016-0384-2

**Published:** 2016-11-03

**Authors:** Maria A. Baturova, Seth H. Sheldon, Jonas Carlson, Peter A. Brady, Grace Lin, Alejandro A. Rabinstein, Paul A. Friedman, Pyotr G. Platonov

**Affiliations:** 1 Department of Cardiology, Clinical Science, Lund University, Lund, SE-221 85 Sweden; 2 University Clinic, St. Petersburg State University, Kadetskaya Line 13-15, St. Petersburg, 199004 Russia; 3 Department of Cardiology, Mayo Clinic, 200 First Street SW, Rochester, Minnesota 55902 USA; 4 Department of Neurology, Mayo Clinic, Mayo West 8B, 200 First Street SW, Mayo Clinic, Rochester, Minnesota 55905 USA; 5 Arrhythmia Clinic, Skåne University Hospital, Lund, SE-221 85 Sweden

**Keywords:** Atrial fibrillation, Ischemic stroke, ECG, Left atrial volume index

## Abstract

**Background:**

Detection of atrial fibrillation after ischemic stroke is challenging due to its paroxysmal nature. We aimed to assess predictors of paroxysmal atrial fibrillation using non-invasive surface ECG and transthoracic echocardiography to select candidates for atrial fibrillation screening.

**Methods:**

Ischemic stroke patients without documented atrial fibrillation (*n =* 110, 67 ± 10 years, 40 female) and a control group of age- and gender-matched patients with history of paroxysmal atrial fibrillation prior to stroke (*n =* 55, 67 ± 10 years, 19 female) comprised the study sample. Using non-invasive ECG monitoring for three weeks, short episodes of paroxysmal atrial fibrillation were detected in 24 of 110 patients (22 %). The standard 12-lead ECG with sinus rhythm at stroke onset was digitally processed and analyzed. Transthoracic echocardiography data were reviewed for these patients.

**Results:**

Atrial fibrillation history was independently associated with P terminal force in lead V 1 > 40 mm*ms (OR 4.04 95 % CI 1.34–12.14, *p =* 0.013) and left atrial volume index (OR 1.08 95 % CI 1.03–1.13, *p =* 0.002; for LAVI > 40 mL/m^2^ OR 6.40 95 % CL 1.47–27.91, *p =* 0.013). Among patients without atrial fibrillation history, no ECG characteristics were predictive of atrial fibrillation detected after stroke. Left atrial volume index remained an independent predictor of atrial fibrillation detected after stroke (OR 1.09 95 % CI 1.02–1.16, *p =* 0.017). A cutoff of <40 mL/m^2^ had an 84 % negative predictive value for ruling out atrial fibrillation on ambulatory monitoring with a sensitivity of 50 % and a specificity of 86 %.

**Conclusion:**

In a *post hoc* analysis, left atrial dilatation assessed by left atrial volume index independently predicted atrial fibrillation after stroke in patients without prior atrial fibrillation history, while the other clinical or ECG markers were not predictive of atrial fibrillation detected early after ischemic stroke.

**Trial registration:**

This study is a *post hoc* analysis from the prospective case-control study registered in December 2011, ClinicalTrials.gov ID: NCT01325545.

## Background

The high prevalence of atrial fibrillation (AF) in ischemic stroke patients is well-documented [1, 2]. Detecting AF after ischemic stroke is important, as anticoagulation therapy, as opposed to aspirin, is hence used to prevent recurrent thromboembolic events. However, paroxysmal AF is often underdiagnosed. Routine diagnostic screening techniques, such as 24-h Holter ECG monitoring, have modest sensitivity for AF detection [3, 4]. Prolonged electrocardiographic (ECG) monitoring increases the detection rate of AF after a stroke [5]. However, the highest detection rate of AF after cryptogenic stroke was reported in stroke patients with implantable cardiac monitors (ICM) [6]. While ICM-based strategy for AF detection is clearly superior to other ECG monitoring techniques, its cost effectiveness is largely affected by proper patient selection, which highlights the need for developing AF risk prediction tools.

Atrial remodeling in patients with known AF can be demonstrated using available non-invasive techniques, such as surface ECG and transthoracic echocardiography (TTE). It is not fully clear whether the same ECG and TTE measures are associated with newly-detected AF after ischemic stroke and may be used to select candidates for more costly and time consuming AF screening.

Frequent supraventricular ectopic activity, including frequent supraventricular premature complexes and supraventricular runs detected by 24-h Holter ECG monitoring, are predictive of AF [7, 8]. A case-control study using ambulatory ECG monitoring for three weeks after a stroke showed that short episodes of asymptomatic AF are common in both patients with cryptogenic stroke and patients with stroke of known cause [9]. Identifying clinical markers predictive of AF after stroke would facilitate AF screening in patients at high risk for developing arrhythmia, and help optimize the use of health care resources.

We aimed to investigate clinical, ECG and TTE characteristics associated with paroxysmal AF in ischemic stroke patients, and to assess whether the same clinical, ECG and TTE parameters are predictive of paroxysmal AF detected using ambulatory ECG monitoring early after ischemic stroke.

## Methods

### Study cohort

The study cohort was recruited from the cohort of ischemic stroke patients treated at Mayo Clinic (Rochester, MN, USA). Patients without history of AF or atrial flutter prior to or at the index stroke event were compared with patients with documented paroxysmal AF by admission with stroke.

Study group of patients without AF history comprised of 110 patients with ischemic stroke of either cryptogenic (*n =* 55) or known cause (*n =* 55) who were previously included in the recently published analysis [9] and who had a surface ECG during sinus rhythm obtained at stroke onset (mean age 67 ± 10 years, 40 female). Using ambulatory ECG monitoring for three weeks (Mobile Cardiac Outpatient Telemetry system - CardioNet, Conshohocken, PA, USA), short AF episodes of median 6 s duration (IQR 6–9) were detected in 24 patients (22 %). All arrhythmic episodes were manually reviewed by a board certified electrophysiologist. The 24 patients with newly detected short AF episodes after stroke (ShortAF Group) were compared to the 86 stroke patients without detected AF (NoAF Group).

Control group was randomly selected from age- and gender-matched patients treated at Mayo Clinic with ischemic stroke with history of paroxysmal AF prior to stroke and sinus rhythm on standard 12-lead ECG at admission (PxAF Group, *n =* 55, 67 ± 10 years, 19 female).

The Mayo Clinic Institutional Review Board approved the research protocol.

### Baseline assessment

Baseline clinical assessment included demographics, body mass index (BMI), comorbid conditions such as cardiac failure, hypertension, ischemic heart diseases, stroke or transient ischemic attack in the past, diabetes and cardiovascular risk profile measured by CHADS_2_ and CHA_2_DS_2_-VASc scales [10].

### ECG analysis

Standard clinical 12-lead ECG recordings with sinus rhythm were obtained at enrollment in all study subjects. Digital signals were extracted and stored in a format readable by MegaCare ECG management system (Siemens-Elema, Stockholm, Sweden. Discontinued). Standard clinical measurements, i.e., P-wave duration, QRS duration, corrected QT interval (using Bazett’s formula), PQ interval and P-wave terminal force in Lead V1 were obtained from the MegaCare system using the University of Glasgow 12-lead ECG analysis algorithm [11]. P-wave terminal force in Lead V1 was defined as duration, in milliseconds, of the terminal (negative) part of the P wave multiplied by its depth in millimeters [12].

P-wave morphology assessment was performed using custom-made software running on MATLAB R2013b (The MathWorks, Inc., Natick, MA, USA) for Linux. The 12-lead ECG was mathematically transformed into orthogonal leads using the pseudo-inverse of the Dower transformation matrix [13]. The orthogonal leads were denoted X (right-left), Y (up-down), and Z (front-back).

In addition to conventional P-wave indices, we analyzed gross morphology of P-waves using an automatic algorithm [14] that classified orthogonal P waves into types as having positive polarity in leads X and Y and negative, biphasic (-/+) or positive polarity in lead Z. Biphasic (+/-) P-waves in inferior lead Y have been defined as an advanced interatrial block with retrograde left atrial activation (IAB).

### Echocardiography

Results of clinically indicated TTE were retrieved from patient medical records. TTE examinations were performed at median 1 day (interquartile range 25–75 % (IQR) -10.9 to 2.9 months) from the stroke event. We assessed the left atrial volume index (LAVI, ml/m^2^), ejection fraction (EF), estimated right atrial pressure using inferior vena cava size and respiratory variation (mm Hg), right ventricular pressure (mm Hg), left ventricular end-systolic and end-diastolic internal dimensions (mm).

### Statistical methods

Clinical characteristics, ECG and TTE parameters were compared between patients in the NoAF Group and patients in the ShortAF Group using chi-square or Fisher’s exact test for categorical variables and Student’s t-test for continuous variables with an approximate normal distribution or alternatively non-parametric tests, as appropriate.

To identify predictors of paroxysmal AF on prolonged ambulatory ECG monitoring, significantly associated covariates were further evaluated in univariate logistic regression models with estimation of odds ratios and likelihood-ratio tests. Factors significantly associated with occurrence of short AF episodes on ECG monitoring in the univariate models were subsequently included in a stepwise multivariate regression analysis with backwards elimination. Predictive accuracy of covariates determined in multivariate logistic regression models was evaluated using receiver operating characteristic (ROC) curve analysis with calculation of positive predictive value, negative predictive value, sensitivity and specificity of the determined parameters.

The group of patients without history of AF prior to or at the index stroke event was compared to patients in the PxAF Group. Univariate logistic regression analysis was performed to identify covariates significantly associated with AF history that were further included in a stepwise multivariate regression analysis with backwards elimination. Threshold values for F-to-enter and F-to-remove were 0.05 and 0.1, respectively.

All analyses were performed using SPSS Statistics 20 (SPSS Inc., Chicago, Illinois, USA). *P-*values of <0.05 were considered significant.

## Results

### Clinical characteristics associated with history of paroxysmal AF in ischemic stroke patients

PxAF patients had a higher proportion of vascular diseases, cardiac failure and higher cardiovascular risk profile measured by CHADS_2_ and CHA_2_DS_2_-VASc scales than patients without AF at baseline (Table 1).

**Table 1:** Clinical, ECG and Echocardiographic characteristics of stroke patients without AF history at stroke onset and patients with history of AF prior to stroke

Variables	Patients without AF history, *n =* 110	Patients with PxAF, *n =* 55	*P* value
Mean age, years^a^	67 ± 10	68 ± 10	0.686
Female, *n* (%)	40 (36)	19 (35)	0.864
BMI^a^	29 ± 5	29 ± 5	0.790
P-wave duration, ms^a^	137 ± 16	145 ± 17	0.003
Patients with P-wave duration > 120 ms, *n* (%)	90 (82)	47 (85)	0.387
PR-interval, ms^a^	172 ± 28	178 ± 35	0.189
P-wave terminal force in lead V1, mm x ms^a^	24 ± 26	35 ± 33	0.020
QRS duration, ms^a^	100 ± 17	107 ± 21	0.021
Corrected QTc interval, ms^a^	430 ± 28	454 ± 34	<0.001
P-wave morphology			0.435
X(+)Y(+)Z(-), *n* (%)	19 (19)	8 (16)	
X(+)Y(+)Z(-/+), *n* (%)	60 (59)	32 (64)	
IAB or X(+)Y(+/-), *n* (%)	2 (2)	3 (6)	
X(+)Y(+)Z(+), *n* (%)	21 (21)	7 (14)	
Left atrium volume index, ml/m^2^	35 ± 12	45 ± 12	<0.001
EF, %^a^	58 ± 12	52 ± 15	0.027
Left ventricular end-systolic internal demension, mm^a^	33 ± 9	37 ± 10	0.033
Left ventricular end-diastolic internal demension, mm^a^	49 ± 7	52 ± 8	0.049
Right atrium pressure, mmHg^a^	6 ± 2	8 ± 5	0.007
Right ventricular systolic pressure, mmHg^a^	34 ± 10	36 ± 12	0.242
Diabetes, *n* (%)	18 (16)	12 (22)	0.399
Hypertension, *n* (%)	84 (76)	41 (75)	0.848
Vascular diseases, *n* (%)	21 (19)	20 (36)	0.021
Cardiac failure, *n* (%)	6 (6)	16 (29)	<0.001
CHADS_2_ score^a^	3.2 ± 0.9	3.5 ± 1.0	0.034
CHA_2_DS_2_-VASc score^a^	4.9 ± 1.5	4.9 ± 1.5	0.028

^a^ - the results are cited as mean value ± standard deviation

Analysis of ECG data showed that P-wave duration, QRS duration, corrected QT interval were longer, and P-wave terminal force in lead V_1_ was greater in PxAF patients than in patients without AF at stroke. The distribution of different P-wave morphologies was similar in both groups. P-wave morphology with positive P-waves in leads X, Y and biphasic P-wave in lead Z was the most prevalent type in our study cohort.

TTE examination revealed that patients with AF history had lower EF, higher right atrium systolic pressure, larger left ventricular end-systolic and end-diastolic internal dimensions and greater LAVI than patients without AF.

However, in multivariate logistic regression analysis, only vascular diseases (odds ratio (OR) 4.10 95 % CI 1.32–12.78, *p =* 0.015), P-wave terminal force in lead V1 greater 40 mm*ms (OR 4.04 95 % CI 1.34–12.14, *p =* 0.013) and LAVI (OR 1.08 95 % CI 1.03–1.13, *p =* 0.002) remained significantly associated with AF prior to stroke.

### Predictors of short AF episodes detected after ischemic stroke in patients without AF history

Clinical, ECG and TTE data are summarized in the Table 2.

**Table 2:** Clinical, ECG and Echocardiographic characteristics of stroke patients without AF history at stroke event

	NoAF Group, *n =* 86	ShortAF Group, *n =* 24	*P* value
Mean age, years^a^	66 ± 10	71 ± 9	0.033
Men, *n* (%)	55 (64)	15 (63)	1.000
BMI^a^	31 ± 23	30 ± 6	0.761
P-wave duration, ms^a^	136 ± 15	143 ± 18	0.232
Patients with P-wave duration > 120 ms, *n* (%)	70 (81)	20 (83)	0.451
PR-interval, ms^a^	175 ± 29	158 ± 22	0.007
P-wave terminal force in lead V1, mm x ms^a^	23 ± 24	28 ± 35	0.394
QRS duration, ms^a^	100 ± 18	100 ± 15	0.962
QTc, ms^a^	430 ± 28	430 ± 28	1.000
PW morphology			0.570
X(+)Y(+)Z(-), *n* (%)	16 (20)	3 (14)	
X(+)Y(+)Z(-/+), *n* (%)	45 (56)	15 (71)	
IAB or X(+)Y(+/-), *n* (%)	2 (3)	0 (0)	
X(+)Y(+)Z(+), *n* (%)	18 (22)	3 (14)	
Left atrium volume index, ml/m^2^	32 ± 10	42 ± 15	0.007
EF, %	58 ± 12	56 ± 13	0.499
Left ventricular S, mm^a^	33 ± 9	35 ± 8	0.496
Left ventricular D, mm^a^	49 ± 7	50 ± 6	0.679
Right atrial pressure, mmHg^a^	6 ± 2	6 ± 2	0.498
Right ventricular systolic pressure, mmHg^a^	33 ± 11	36 ± 7	0.490
Diabetes, *n* (%)	17 (20)	1 (4)	0.115
Hypertension, *n* (%)	66 (76)	18 (75)	1.000
Ischemic heart disease, *n* (%)	17 (20)	4 (17)	1.000
Cardiac failure, *n* (%)	4 (5)	2 (9)	0.604
CHADS_2_ score^a^	3.2 ± 0.9	3.2 ± 0.9	0.996
CHA_2_DS_2_-VASc score^a^	4.3 ± 1.5	4.5 ± 1.4	0.579

^a^- the results are cited as mean value ± standard deviation

Patients with AF detected on ECG monitoring were older than patients without detected AF, with no differences in sex, BMI, cardiovascular comorbidities or cardiovascular risk profile measured by CHADS_2_ and CHA_2_DS_2_-VASc scales. These patients had greater LAVI than patients without AF, and there were no differences in other ECG (including P-wave morphology) and TTE characteristics.

In univariate regression analysis, detection of short AF episodes after stroke was associated only with age and LAVI. However, LAVI remained the only independent predictor of AF in multivariate regression analysis (Table 3).

**Table 3:** Covariates associated with short AF episodes on ambulatory ECG monitoring detected in patients without prior AF history

	Univariate regression model			Multivariate regression model (adjusted for age)		
Variables	OR	95 % CI	*P* value	OR	95 % CI	*P* value
^a^LAVI	1.08	1.01–1.15	0.017	1.08	1.01–1.15	0.017
^a^LAVI > 40 ml/m^2^	6.40	1.47–27.91	0.013	6.40	1.47–27.91	0.013
P-wave duration	1.03	1.00–1.06	0.082			
QRS duration	1.00	1.00–1.03	0.961			
Corrected QT interval	1.00	1.00–1.02	0.988			
PTF	1.00	1.00–1.00	0.393			
EF	1.00	1.00–1.03	0.493			
Left ventricle end-diatolic dimension	1.02	1.00–1.12	0.672			
Left ventrical end-systolic dimension	1.03	1.00–1.10	0.488			
Right atrium pressure	1.11	0.82–1.52	0.492			
Age	1.05	1.00–1.11	0.037			
Hypertension	0.91	0.32–2.60	0.859			
Ischemic heart diseases	0.81	0.25–2.69	0.733			
Cardiac failure	1.86	0.35–10.85	0.488			

^a^- multivariate analysis was performed separately for LAVI as continuous variable and for LAVI > 40 ml/m^2^

**Fig. 1:**
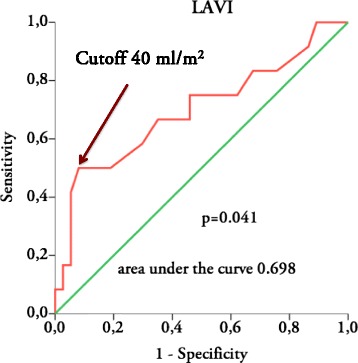
ROC curve for LAVI to detect short episodes of AF on ambulatory ECG monitoring

The area under the ROC curve values for LAVI as an indicator of the short AF episodes detected by ambulatory ECG monitoring was 0.698, *p =* 0.041 (Fig. 1). A cutoff of <40 mL/m^2^ had a positive predictive value of 55 % and an 84 % negative predictive value for ruling out AF on ambulatory monitoring, with sensitivity 50 % and specificity 86 %.

## Discussion

Detecting AF after ischemic stroke is challenging due to its paroxysmal nature. Studies with implantable devices have shown that most AF episodes are asymptomatic [15, 16], and thus AF often remains undetected. Routine ECG monitoring strategies have moderate sensitivity in AF detection, and there is therefore a need to find a simple non-invasive tool to identify stroke survivors who would benefit more from continuous screening for AF.

Our main finding is that left atrial dilatation measured by LAVI is the strongest independent predictor of subsequent detection of short AF episodes during prolonged ECG monitoring in patients without AF at stroke onset. ECG data, including P-wave morphology, and underlying comorbidities have limited value for predicting paroxysmal AF after ischemic stroke. In patients with known paroxysmal AF, however, a negative terminal deflection in V1 was a strong indicator of arrhythmia.

The novelty of our finding is that the presence of electrocardiographic abnormalities in patients with known AF, but not in those without history of AF, suggests that initially there is development of subtle structural changes seen by TTE (increased LAVI), and electrocardiographic abnormalities are only seen later.

### Short episodes of paroxysmal AF

In patients without AF history at stroke onset, we found very short episodes of paroxysmal AF during 3 weeks of ECG monitoring. It is still under discussion whether ultra-short AF episodes of less than 30 s have the same risk of thromboembolic complications as manifested AF [17]. However, it has been shown that supraventricular runs and high supraventricular ectopic activity are predictive of AF occurrence [7, 8]. In ischemic stroke patients, premature atrial complexes that occur more frequently than 4 per hour and atrial runs that exceed 5 complexes were associated with occurrence of paroxysmal AF [18]. Studies with loop recorders implanted for AF screening after ischemic stroke reported detection of AF lasting 2 min or longer on average 48–68 days after implantation [19, 20]. While short episodes of paroxysmal AF were common for our ischemic stroke cohort who underwent ambulatory ECG monitoring for 3 weeks, the monitoring time was perhaps not long enough to reveal full incidence of underlying asymptomatic AF in this stroke cohort. Short episodes of paroxysmal AF may be considered as “surrogates” for prolonged AF that should be used for identifying stroke patients who would benefit from continuous screening for AF.

It is uncertain whether short episodes of AF indicate the need for anticoagulation therapy. The TRENDS study in patients with implantable devices showed that AF burden exceeding 5.5 h during any of the preceding 30 days appeared to double the thromboembolic risk [15]. However, in the ASSERT study in patients with implantable devices it has been shown that most patients who had stroke while being monitored did not have AF at the time and 30 days prior to the stroke onset [16].

As reported recently, early anticoagulation therapy for incident AF and withdrawal of anticoagulation therapy after arrhythmia-free periods do not improve outcomes in patients with implantable devices as compared with conventional management [21]. Additional studies are needed to investigate the benefit of anticoagulant therapy in patients with short asymptomatic episodes of paroxysmal AF.

### Atrial remodeling predisposing to atrial fibrillation

#### Left atrial volume index

Left atrial dilatation measured as increase in LAVI may be a marker of underlying structural changes in the atrium leading to the development of AF in patients without advanced cardiovascular disorders. Notably, it has been shown that LAVI was associated with first-ever ischemic stroke in patients without previous AF. [22] One possible explanation for this association is that blood stasis and thrombus formation may occur more often in a left atrium of increased size even when AF is not present [23]. Another possible explanation is that these patients actually have undetected paroxysmal AF that was not present at the time of stroke. Furthermore, inflammation and structural changes due to underlying comorbidity may be more important than atrial fibrillation in the development of atrial myopathy. The CHA_2_DS_2_VASC score increases stroke risk even in the absence of known AF [24].

Increased LAVI reflects remodeling of left atrium due to pressure or volume overload [25] and correlates with the extent of left atrial fibrosis [26]. Both atrial remodeling and atrial fibrosis are pathological changes associated with the development of AF.

It has been shown that LAVI has a high diagnostic accuracy for AF in hypertensive patients with historical paroxysmal AF [27]. This was confirmed in our study for stroke patients with known AF. We have also shown that even in patients without history of AF, LAVI independently predicted AF detected by ambulatory ECG monitoring early after ischemic stroke, and can be used in routine clinical practice as a valuable index for selecting patients for continuous ECG monitoring for AF detection. LAVI < 40 mL/m^2^ has a high negative predictive value for ruling out short AF episodes on ambulatory ECG monitoring. This approach may decrease the number of patients undergoing ambulatory ECG monitoring after stroke and consequently reduce costs of medical care for this patient population. Conversely, patients with LAVI > 40 mL/m^2^ are more likely to have episodes of asymptomatic AF and should be screened for AF more thoroughly.

In our study, LAVI was associated with history of AF independently from other TTE characteristics, and was higher in patients with history of AF than in patients with short AF episodes. While the difference was not significant (perhaps due to the small number of patients with detected AF after stroke), we observed a trend of gradual LAVI increase from its lowest value in patients without any AF, to intermediate volume in patients with short AF episodes, and highest LAVI in patients with a history of AF. This trend may reflect the underlying progression structural changes in the left atrium in patients who develop AF.

#### ECG data

In order to assess ECG predictors of AF detected shortly after ischemic stroke, we used ECG characteristics that were reported to be associated with AF in earlier studies.

One of the most studied markers of atrial conduction is P-wave duration. P-wave prolongation reflects atrial remodeling predisposing to occurrence of AF. In the Framingham Heart Study, the prolongation of P-wave duration predicted the development of AF during long-term follow-up in an elderly community-based cohort [28]. In hypertensive patients, prolonged P-wave duration was associated with AF incidence during 25 ± 3 months [29]. In our study, the majority of patients initially had prolonged P-wave duration, but no association was found between P-wave duration and AF on ambulatory ECG monitoring. These findings are in line with an earlier report of absent association between P-wave prolongation and incident AF among patients with advanced congestive heart failure and prolonged P-waves at baseline in the MADIT-II study [14].

Sinus P-waves with biphasic morphology in the sagittal plane (right precordial leads or orthogonal lead Z) quantified as increase of negative terminal force in lead V_1_ (e.g. P terminal force in lead V1) was predominantly found in the elderly [30] and in patients with a history of AF [31] or structural heart disease [32]. The Atherosclerosis Risk in Communities study found that P terminal force in lead V1 greater than 4000 μV * ms was associated with an increased risk of AF. [33] In our study, P terminal force in lead V1 greater than 40 mm*ms was independently associated with history of AF, which is in line with previously reported data.

In summary, while ECG characteristics in patients with established AF and documented arrhythmia prior to stroke expectedly had prolonged P-waves and more prominent P terminal force in lead V1, none of these ECG characteristics was independently predictive of paroxysmal AF detected in the cohort of patients without prior AF history. While patients with short AF episodes had longer P-wave duration and greater P terminal force in lead V1, the difference did not reach statistical significance. The apparent lack of association between AF detection after stroke and P-wave abnormalities is perhaps due to the fact that most patients included in our study had underlying cardiovascular comorbidities and atrial fibrosis affecting P-wave duration and morphology. Another explanation for this is that P-wave abnormalities are a later finding in atrial myopathy disease progression than LAVI enlargement, and our patients were relatively early in their disease progression.

It is possible that we did not have enough patients with short AF episodes detected after stroke in our study, thus possibly undermining our ability to identify an association with P-wave duration and morphology. However, LAVI as a marker of structural left atrial remodeling was significantly associated with history of AF and had strong predictive value for incident AF, thus demonstrating its superiority over ECG indices. Indeed, future studies could determine whether increased LAVI alone may predict which patients would fulfill the indications for initiating oral anticoagulation therapy.

### Study limitations

The main limitation of our study is the relatively small number of patients who had short AF episodes on ambulatory ECG monitoring; this small patient population may affect the interpretation of negative findings of the lack of predictive value of ECG indices for incident AF. Our analysis is also affected by limitations inherent in the study’s retrospective design. We used cinically indicated TTE data obtained from patients’ medical records. While most patients had TTE done at the time of admission with ischemic stroke (median time 1 day after stroke), we can not completely rule out the impact of TTE timing in relation to stroke on its predictive value with regard to new-onset AF during follow-up.

Consequently, our findings should be considered hypothesis-generating and need to be independently reproduced in further studies.

## Conclusion

LAVI is the strongest independent predictor of paroxysmal AF detected after ischemic stroke. LAVI may be considered as an early marker of asymptomatic AF in stroke patients without history of AF and advanced structural changes in the heart, which may help identify patients who would benefit from intensive monitoring for AF detection. Most stroke patients with LAVI < 40 mL/m^2^ are less likely to develop paroxysmal AF on prolonged ambulatory ECG monitoring.

## Abbreviations

AF: Atrial fibrillation
BMI: Body mass index
ECG: Electrocardiographic
EF: Ejection fraction
IQR: Interquartile range 25–75 %
LAVI: Left atrial volume index
OR: Odds ratio
Px: Paroxysmal
ROC: Receiver operating characteristic
TTE: Transthoracic echocardiography
